# Biotransformation of tobacco-derived Z-abienol into precursors of ambrox by newly identified *Acinetobacter tjernbergiae* LSC-2

**DOI:** 10.3389/fmicb.2025.1581788

**Published:** 2025-05-14

**Authors:** Gaolei Xi, Wenyuan Qi, Aamir Rasool, Yongzhen Zhao, Qingfu Wang, Liuke Zhang, Haoyang Chen, Xinlong Zhang, Shen Huang, Zhifi Chen

**Affiliations:** ^1^Technology Center for China Tobacco Henan Industrial Limited Company, Zhengzhou, Henan, China; ^2^Key Laboratory of Biotechnology in Tobacco Industry, Zhengzhou University of Light Industry, Zhengzhou, China; ^3^Institute of Biochemistry, University of Balochistan, Quetta, Pakistan; ^4^Jamil-ur-Rahman Center for Genome Research, Dr. Panjwani Center for Molecular Medicine and Drug Research, International Center for Chemical and Biological Sciences, University of Karachi, Karachi, Pakistan

**Keywords:** Z-abienol, *Acinetobacter tjernbergiae*, biotransformation, diterpene degradation, oxidoreductases, fragrance industry, microbial fermentation

## Abstract

Z-abienol is a labdane diterpene present in tobacco leaves and is a key precursor for producing valuable aroma compounds such as ambrox. This study aimed to identify and characterize a bacterial strain that can efficiently degrade Z-abienol through microbial fermentation. The strain LSC-2 isolated from fresh tobacco leaves was identified as *Acinetobacter tjernbergiae* based on its morphological features and 16S rDNA phylogenetic analysis. Results of fermentation optimization experiments showed that the highest degradation efficiency of LSC-2 (69.3%) was achieved under the following conditions: 1 mg/mL Z-abienol, 0.5 mg/mL urea as the nitrogen source, pH 7, 30°C, and 150 rpm over 4 days. Whole-genome sequencing and functional annotation revealed that oxidoreductases, especially those from the auxiliary activity enzyme family, play a critical role in Z-abienol degradation. High-performance liquid chromatography and gas chromatography–mass spectrometry analysis confirmed the biotransformation of Z-abienol into various intermediates, including sclareol (211.3 μg/mL), scalaral (89.5 μg/mL), and amberonne (57.0 μg/mL). These intermediates have significant industrial applications, particularly in fragrance, pharmaceutical, and cosmetic industries. Sclareol serves as a key precursor in ambrox synthesis, a widely used fixative in high-end perfumery, whereas scalaral and amberonne enhance the aroma in tobacco and flavor formulations. The findings of this study provide valuable insights into the microbial degradation of Z-abienol, which will help develop a sustainable approach to producing bio-based fragrance compounds. Future studies should focus on enzymatic mechanisms and metabolic engineering strategies to improve the efficiency of biotransformation.

## 1 Introduction

Tobacco is a cash crop that acts as a source of thousands of chemical compounds, including secondary metabolites responsible for its characteristic flavor, aroma, and physiological effects (Huang et al., 2024; Peng et al., 2025; Wang Y. et al., 2024). Recently, the extraction of bioactive compounds from plants and animals has gained significant research interest (Lodi et al., 2025; Suleria et al., 2016; Wang R. et al., 2024; Zou et al., 2021). cis-Abienol, also known as Z-abienol, is predominantly found in tobacco plants (*Nicotiana tabacum*) and balsam fir (*Abies balsamea*). It acts as a precursor for the semi-synthesis of amber compounds, which are sustainable replacements for ambergris and are widely used in fragrance, medicine, condiment, and traditional Chinese medicine industries (Cheng et al., 2020; Lin et al., 2024; Ncube et al., 2020). Ambergris and its derivatives, such as ambroxide and ambroxan, have various therapeutic properties with potential health benefits. These substances show significant cytotoxic activity against various cancer cell lines, including liver, colon, lung, and breast cancers (Shen et al., 2007). In addition, ambrein has been identified as an effective aphrodisiac, enhancing sexual behavior in animal models by influencing hormonal activity (Taha et al., 1995). Moreover, ambergris shows anti-inflammatory properties by inhibiting human neutrophil function and reducing inflammatory responses (Shen et al., 2007). Its antidiabetic effects have been observed in diabetic rats, as it lowers blood glucose levels by enhancing glucose utilization (Taha, 1991). Furthermore, ambergris shows analgesic and antinociceptive effects and effectively reduces pain sensitivity in rodent models (Taha, 1992).

The triterpenoid (+)-ambrein, a rare compound that is the major component of ambergris, is a waxy substance produced in the intestines of 1% of the sperm whale (*Physeter macrocephalus*) population, which hardens and transforms after exposure to seawater and sunlight (Du et al., 2020; Lin et al., 2024; Martins et al., 2015). (+)-Ambrein can be oxidatively degraded into several fragrant compounds, among which ambrox is one of the most valuable compounds in the perfume industry (Diao et al., 2023). Currently, the market demand for ambrox is met through chemical synthesis and enzyme-catalyzed transformation of precursors such as sclareol, cis-abienol, and labdanolic acid (Eichhorn et al., 2018; Moser et al., 2020; Yang et al., 2016) or via homofarnesol (Eichhorn et al., 2018). The annual use of ambrox, the most valuable substitute for ambergris, is estimated to exceed 100 tons (Eichhorn et al., 2018; Read, 2013). The transformation of cis-abienol into ambrox has advantages such as fewer reaction steps and higher selectivity and yield (Eichhorn et al., 2018; Zhang et al., 2022).

On the other hand, researchers are also focused on reducing the content of harmful substances present in tobacco and enhancing the production of neutral aroma-enhancing compounds (NAECs) via several strategies, such as tobacco leaf blending technology and addition of microbial spices. Fermentation of reconstituted tobacco leaves (RCTLs) with *Klebsiella variicola*, along with native microorganisms, has been shown to enhance NAECs and nicotine (Huang et al., 2024). Furthermore, the addition of microorganisms in the fermentation process of RCTLs has been shown to reduce pungent odors and eliminate bitter tastes (Li et al., 2021; Lu et al., 2014), decrease the content of tar and other harmful substances (Huang S. et al., 2022; Potts et al., 2010), enhance combustibility, and reduce air pollution (Brown and Cheng, 2014; Wang et al., 2005; Zeng et al., 2023).

In this study, we aimed to screen, isolate, and characterize a microbial strain that converts tobacco-derived compounds into valuable compounds. As a result, we identified *Acinetobacter tjernbergiae* that biodegrades Z-abienol into scalaral and amberonne, which serve as precursors to ambrox. In addition, this strain can be used in the fermentation of RCTLs to enhance the sensory attributes of cigarettes.

## 2 Materials and methods

### 2.1 Sample collection and isolation and selection of LSC-2 strain

Tobacco leaves of the Basma variety and soil samples were obtained from Yunnan Spice Tobacco Co., Ltd. The leaves were collected from the upper, middle, and lower sections of the plants. Isolation and enrichment of the bacterial strain were performed as previously described (Huang et al., 2024). Briefly, 1 g of tobacco leaves or soil was suspended in 100 mL of sterile water and was incubated at 30°C for 1 h while being agitated. A 150-μL aliquot of the suspension was transferred to Luria–Bertani broth and further incubated at 30°C with 150 rpm for 6 h to enrich microbial populations. Subsequently, 2% (v/v) of the enriched culture was inoculated in a selective medium containing 1% (w/v) Z-abienol as the sole carbon source (medium composition referenced from the aforementioned study) and incubated under the same conditions for 48 h for the screening of Z-abienol-degrading bacteria.

The mixed culture that showed Z-abienol degradation activity was serially diluted (10^−5^ to 10^−9^) and plated on selective agar, and individual colonies were purified. Gas chromatography–mass spectrometry (GC-MS) analysis identified the strain LSC-2 as the most efficient Z-abienol degrader and confirmed its metabolic capability.

### 2.2 Determination of the growth curve of the LSC-2 strain

The *A. tjernbergiae* LSC-2 strain was inoculated in 1% (w/v) Z-abienol-supplemented medium with an inoculum size of 2% (v/v), in accordance with the previously mentioned protocols (Huang S. et al., 2022). The culture was incubated at 30°C with shaking at 150 rpm. Optical density at 600 nm (OD600) was measured at 6-h intervals to monitor bacterial growth.

### 2.3 Optimization of the Z-abienol degradation potential of the LSC-2 strain

The Z-abienol degradation rate of the LSC-2 strain was optimized by evaluating the effects of the following parameters: (i) Z-abienol concentration, (ii) temperature, (iii) carbon source, (iv) nitrogen source, (v) nitrogen source concentration, (vi) rotation speed, and (vii) pH. Each group of experiments was conducted in triplicate.

The optimal Z-abienol concentration was determined among 1, 2, 4, 6, and 8 mg/mL in a selective medium as a carbon source. The experiment was conducted under the following conditions: pH 7, inoculum 100 μL, temperature 30°C, incubation time 96 h, and a final fermentation broth volume of 5 mL, incubated in a rotary shaker at 150 rpm. To determine the optimal temperature for Z-abienol degradation, the LSC-2 strain was grown in the selective medium under identical conditions, except for temperature, which was varied as follows: 20°C, 25°C, 30°C, 35°C, and 40°C. The effect of different carbon sources, including glucose, maltose, sucrose, and cyclodextrin, on Z-abienol degradation was also evaluated. Furthermore, various nitrogen sources, such as ammonium sulfate, urea, soybean, xanthopterin, sodium nitrate, and potassium nitrate, were tested to determine the most effective among them. The effect of pH on the degradation rate of Z-abienol was assessed by growing the LSC-2 strain in the selective medium with pH adjusted to 5, 6, 7, 8, and 9. Furthermore, the optimal rotation speed of the shaking incubator was identified among different speeds, namely 100, 150, 200, 250, and 300 rpm.

### 2.4 GC-MS analysis of Z-abienol biodegradation products

The products of Z-abienol biodegradation were extracted from the supernatant of the fermented selective medium. The supernatant was obtained by centrifuging 5 mL of the fermented selective medium at 1,000 rpm for 10 min. It was then mixed with 5 mL of dichloromethane (99% pure), and the extraction process was repeated three times. The organic phase was then separated and concentrated using a rotary evaporator set at 60°C, and the volume was reduced to 1 mL. The concentrated extract was subsequently filtered through a 0.22-μm filter.

GC-MS analysis was performed using an HP-5MS (Agilent Technologies) (5% phenyl methyl siloxane) capillary column (30 m × 0.25 mm × 0.25 μm). The injection temperature was set at 280°C, with helium (He) as the carrier gas at a flow rate of 1 mL/min. The split ratio was 5:1, and the sample injection volume was 1 μL. The temperature program was initiated at 50°C, held for 3 min, and then increased to 130°C at a rate of 6°C/min, where it was maintained for 3 min. Temperature was then increased again to 180°C at a rate of 4°C/min and held for 2 min before a final temperature of 280°C was reached at 3°C/min, which was maintained for 5 min. The GC-MS analysis was conducted in full-scan mode.

The MS conditions included a transfer line temperature of 280°C, an electron ionization energy of 70 eV, a multiplier voltage of 1,450 V, and a mass scan range of 50–700 amu. Ion source temperature was set at 230°C, while quadrupole temperature was maintained at 150°C.

Finally, Z-abienol biodegradation products were identified by comparing the obtained mass spectra with those in the NIST library database. Dichloromethane was used as an internal standard to quantify Z-abienol biodegradation products.

### 2.5 HPLC analysis for the estimation of Z-abienol biodegradation rate

The fermented selective medium supplemented with Z-abienol (test group) and the one without Z-abienol supplementation (control group) were compared to estimate the Z-abienol biodegradation rate. A 15 mL sample from each group was collected for analysis. An equal volume of dichloromethane (99% pure) was added to each sample, followed by vortex mixing for 10 min. This extraction step was repeated three times.

All organic extracts were combined and dried using a rotary evaporator set at 60°C. The resulting pellet was dissolved in 1 mL of high-performance liquid chromatography (HPLC)-grade methanol.

HPLC analysis was carried out using a column with the following specifications: 4.6 mm × 250 mm × 5 μm. The mobile phase consisted of methanol (90%), water (8%), and acetate solution (2%). The mixture was filtered through a 0.22-μm organic microfilter and sonicated to remove bubbles. The column temperature was maintained at 30°C, and detection was carried out at a wavelength of 237 nm using a diode array detector (DAD) detector (Guo et al., 2024). The mobile phase flow rate was 1 mL/min, and the injection volume was 5 μL.

Z-abienol (99% pure) was used as a standard to quantify the remaining Z-abienol in the medium, and its degradation rate was estimated using the following formula:


$$\begin{array}{l} \text{Degradation of Z-abienol =}\\ \;\left(\frac{\text{Initial concentration-Residual concentration}}{\text{Initial concentration}}\right)\;\times \text{ 100} \end{array}$$

### 2.6 Characterization of the LSC-2 strain

#### 2.6.1 Morphological characterization of the LSC-2 strain with SEM

For morphological characterization, 1 mL of bacterial suspension was centrifuged at 8,000 rpm for 5 min, and the supernatant was discarded. The pellet was resuspended in 1 mL of precooled 2.5% glutaraldehyde fixative and incubated at 4°C for 4 h for fixation. After fixation, the sample was washed three times with 0.1 M phosphate buffer (pH 7.2–7.4). Dehydration was carried out using 100% ethanol. This step was repeated twice, each lasting 15–20 min. Subsequently, the sample was treated twice with isoamyl acetate to replace ethanol, with each treatment lasting 20 min.

The supernatant was removed following centrifugation, which resulted in a small amount of liquid. A small sample of the liquid was placed on clean slides and dried overnight in an oven. The dried slides with biological samples were then affixed to the sample holder using conductive adhesive (carbon tape). Finally, the samples were coated with a thin layer of gold and then observed under a scanning electron microscope for morphological analysis.

#### 2.6.2 Identification of the LSC-2 strain through 16S rDNA sequencing

The *A. tjernbergiae* strain LSC-2 was identified and characterized based on colony morphology, cell shape, Gram staining, and scanning electron microscopy (SEM).

For molecular identification, genomic DNA was extracted using a bacterial genomic DNA extraction kit (QIAGEN). The universal primers used for 16S rDNA amplification were 27F (5′-AGA GTT TGA TCC TGG CTC AG-3′) and 1492R (5′-GGT TAC CTT GTT ACG ACT T-3′). Polymerase chain reaction (PCR) was carried out in a reaction volume of 25 μL, containing 0.2 μL of Taq polymerase (5 U/μL), 0.5 μL of upstream primer (10 μmol/L), 0.5 μL of downstream primer (10 μmol/L), 2.5 μL of 10 × buffer, 0.5 μL of DNA template (50 ng/μL), 0.5 μL of dNTP mix (10 μmol/L), and 19.8 μL of ddH_2_O. The PCR-amplified 16S rDNA product was sent to Shanghai Parsono Biotechnology Co., Ltd. (Shanghai, China) for sequencing. The obtained sequence was compared with the sequences available in the NCBI GenBank database using BLAST (https://blast.ncbi.nlm.nih.gov/Blast.cgi) to determine the closest related species. Phylogenetic analysis was carried out using MEGA software (version 7.0, Mega Limited, Auckland, New Zealand), and a maximum-likelihood phylogenetic tree was constructed with 1,000 bootstrap replicates to confirm the strain's taxonomic position (Huang S. et al., 2022).

The 16S rDNA sequence of *A. tjernbergiae* LSC-2 strain was submitted to the NCBI Sequence Read Archive (https://www.ncbi.nlm.nih.gov) with the accession number PV065465.

### 2.7 Gene function prediction of bacterial strains

The whole-genome shotgun sequencing strategy was used to construct the LSC-2 gene library of the genomic DNA of the *A. tjernbergiae* LSC-2 strain. High-throughput sequencing was conducted using the Illumina NovaSeq platform (Illumina, San Diego, CA, USA) at Shanghai Paralon Biotechnology Co., Ltd. (Shanghai, China). Sequencing reads were quality-checked using FastQC (Babraham Bioinformatics) and preprocessed with Cutadapt (SciLifeLab) to remove adapters and low-quality reads. Genome assembly was carried out using MEGAHIT (github), followed by annotation and functional analysis.

To identify Carbohydrate-Active enZymes (CAZymes) in the *A. tjernbergiae* LSC-2 genome, hmmscan (European Bioinformatics Institute) software was used to scan the genome against the Carbohydrate-Active enZYmes (CAZy) database.

## 3 Results and discussion

### 3.1 Screening and morphological and molecular characterization of Z-abienol-degrading bacteria

**Figure 1:**
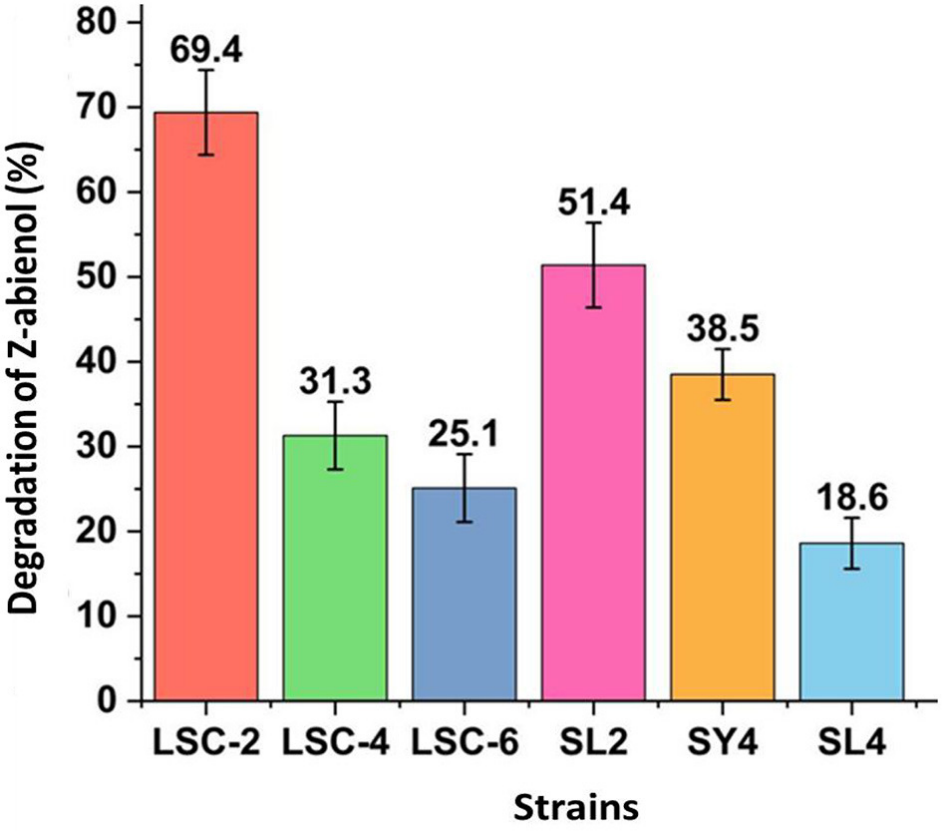
Screening of *Z*-abienol-degrading bacterial strains. Z-abienol degradation efficiency of different bacterial strains was assessed by determining the proportion of Z-abienol degraded. LSC-2 showed the highest metabolic capacity among the tested strains, degrading 69.4% of Z-abienol. In comparison, SL2 degraded 31.3%, and SY4 degraded 38.5%. Error bars represent the variability observed across the experimental triplicate. All data are presented as mean values ± standard deviation (SD, *n* = 3).

Screening of Z-abienol-degrading bacteria revealed multiple strains with distinct degradation capacities. As shown in Figure 1, the LSC-2 strain showed the highest degradation efficiency by metabolizing 69.4% of Z-abienol, which reflects its robust enzymatic activity. The SL2 strain also showed notable degradation performance, removing 51.4% of Z-abienol. Other strains showed intermediate degradation rates: SY4 degraded 38.5% of the compound, LSC-4 31.3%, and LSC-6 25.1%. Conversely, the SL4 strain showed minimal degradation capability with only 18.6% of Z-abienol removal, which indicates deficient metabolic pathways.

Variations in Z-abienol degradation efficiency between LSC-2, LSC-4, LSC-6, SL2, SY4, SL4, and SY4 bacterial strains are attributable to differences in their enzymatic profiles, catalytic activities, and gene expression levels (Berasategui et al., 2017; Janssen et al., 2005).

Evolution plays a crucial role in developing enzymatic diversity, which enables bacteria to resist toxic compounds (Janssen et al., 2005; Peng et al., 2024). Variations in gene expression can result from differences in regulatory mechanisms, environmental conditions, or genetic mutations, leading to diverse metabolic capabilities between strains. For instance, bacterial degradation of xenobiotic compounds has been reported to be influenced by these factors (Egorov et al., 2018; Janssen et al., 2005; Mishra et al., 2022).

**Figure 2:**
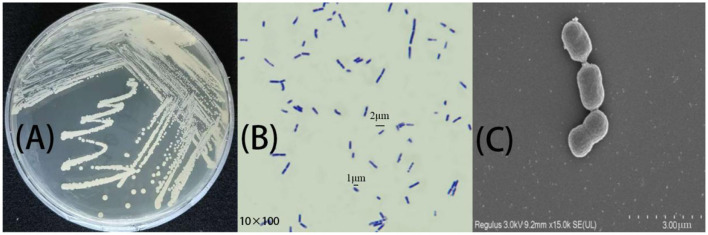
Morphological characteristics of *A. tjernbergiae* LSC-2. **(A)** The colony morphology of the LSC-2 strain grown on an agar plate shows smooth circular creamy white colonies. **(B)** Gram staining results under a light microscope (1,000× magnification) indicate Gram-negative short rod-shaped bacterial cells. **(C)** Scanning electron microscopy (SEM) image at 15,000× magnification, revealing the characteristic coccobacillary morphology of *Acinetobacter* LSC-2.

Morphological characterization of the *A. tjernbergiae* LSC-2 strain was carried out through colony morphology analysis, Gram staining, and SEM. As shown in Figure 2A, the strain formed round white colonies with smooth and opaque surfaces after 48 h of incubation on a solid medium. The colonies showed neat and well-defined edges.

Gram staining results (Figure 2B) confirmed that *A. tjernbergiae* LSC-2 is a Gram-negative bacterium with a short rod-shaped morphology. The SEM micrograph (Figure 2C) provided a more detailed observation that *A. tjernbergiae* LSC-2 cells are non-flagellated short rod-shaped structures measuring ~6–8 μm × 15–18 μm.

Studies investigating the role of *Acinetobacter* spp. in various environmental and biotechnological applications also reported a smooth round colony morphology of *Acinetobacter* strains (Dahal et al., 2023; Gielnik et al., 2019). Moreover, the dimensions observed under the scanning electron microscope were consistent with earlier descriptions of *Acinetobacter* strains isolated from various environments (Touchon et al., 2014).

**Figure 3:**
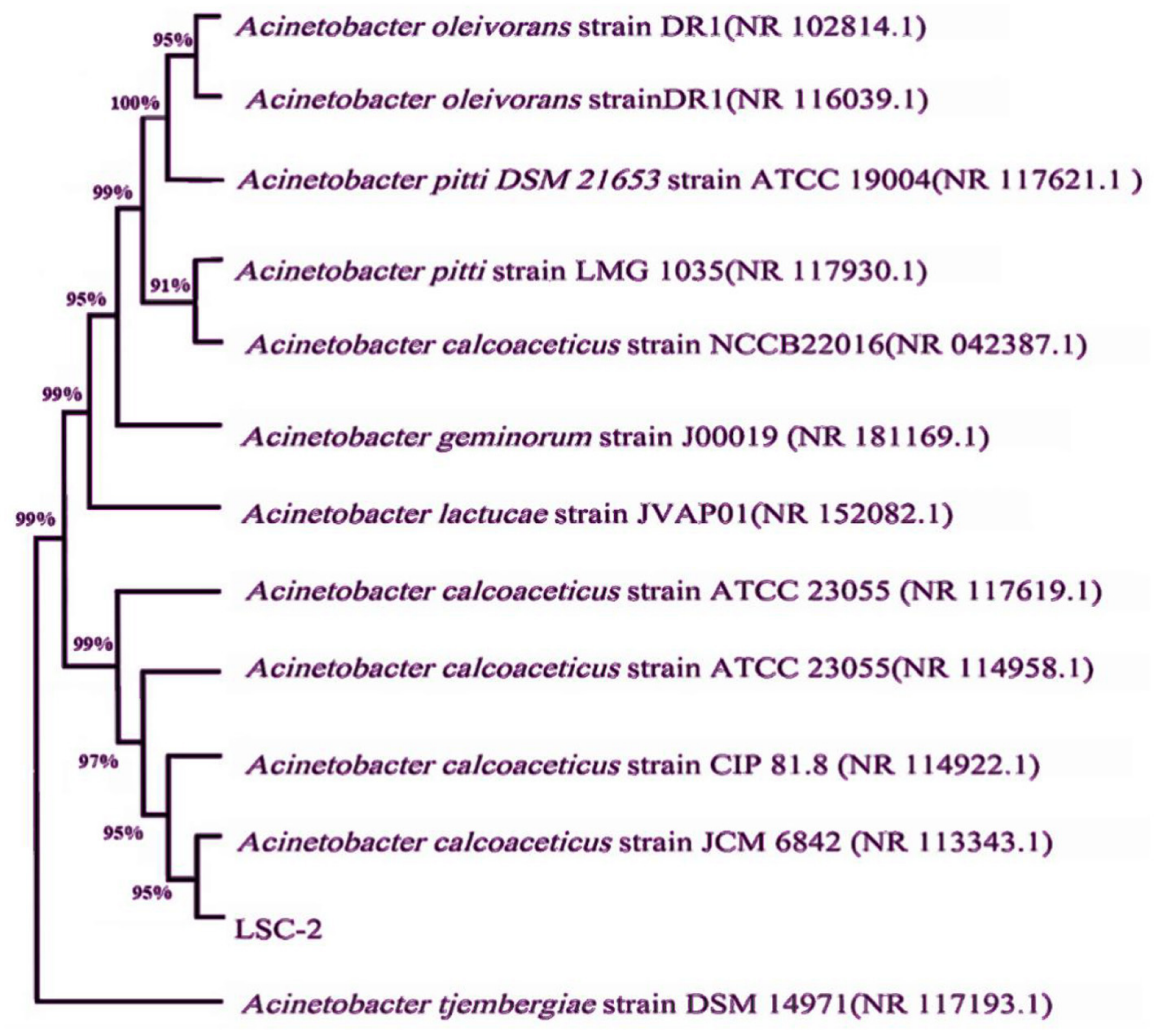
Phylogenetic tree constructed from the 16S rDNA sequence of the LSC-2 strain. The phylogenetic tree shows the evolutionary relationship of LSC-2 with closely related *Acinetobacter* species. Bootstrap values are indicated at the nodes, representing the confidence level of the branching points. The clustering pattern suggests that LSC-2 is closely related to *Acinetobacter tjernbergiae*, supporting its taxonomic placement within the *Acinetobacter* genus.

The molecular and taxonomic characterization of the LSC-2 strain was carried out through 16S rDNA sequencing. The 16S rDNA sequence was aligned against the NCBI GenBank database using BLAST, which showed that LSC-2 shares more than 90% sequence identity with members of the *Acinetobacter* genus, indicating a close evolutionary relationship (Figure 3). Phylogenetic tree analysis showed that LSC-2 clusters closely with *A. tjernbergiae*, suggesting its affiliation with this species and the *Acinetobacter* clade (Figure 3). Furthermore, previous studies have extensively carried out phylogenetic analyses using 16S rDNA for bacterial taxonomy and species delineation (Nemec et al., 2021; Touchon et al., 2014).

Morphological observations, molecular identification, and phylogenetic analysis revealed that the LSC-2 strain belongs to the *A. tjernbergiae* species and the *Acinetobacter* genus.

### 3.2 Growth profile, Z-abienol degradation, and metabolite formation of *Acinetobacter tjernbergiae* LSC-2

**Figure 4:**
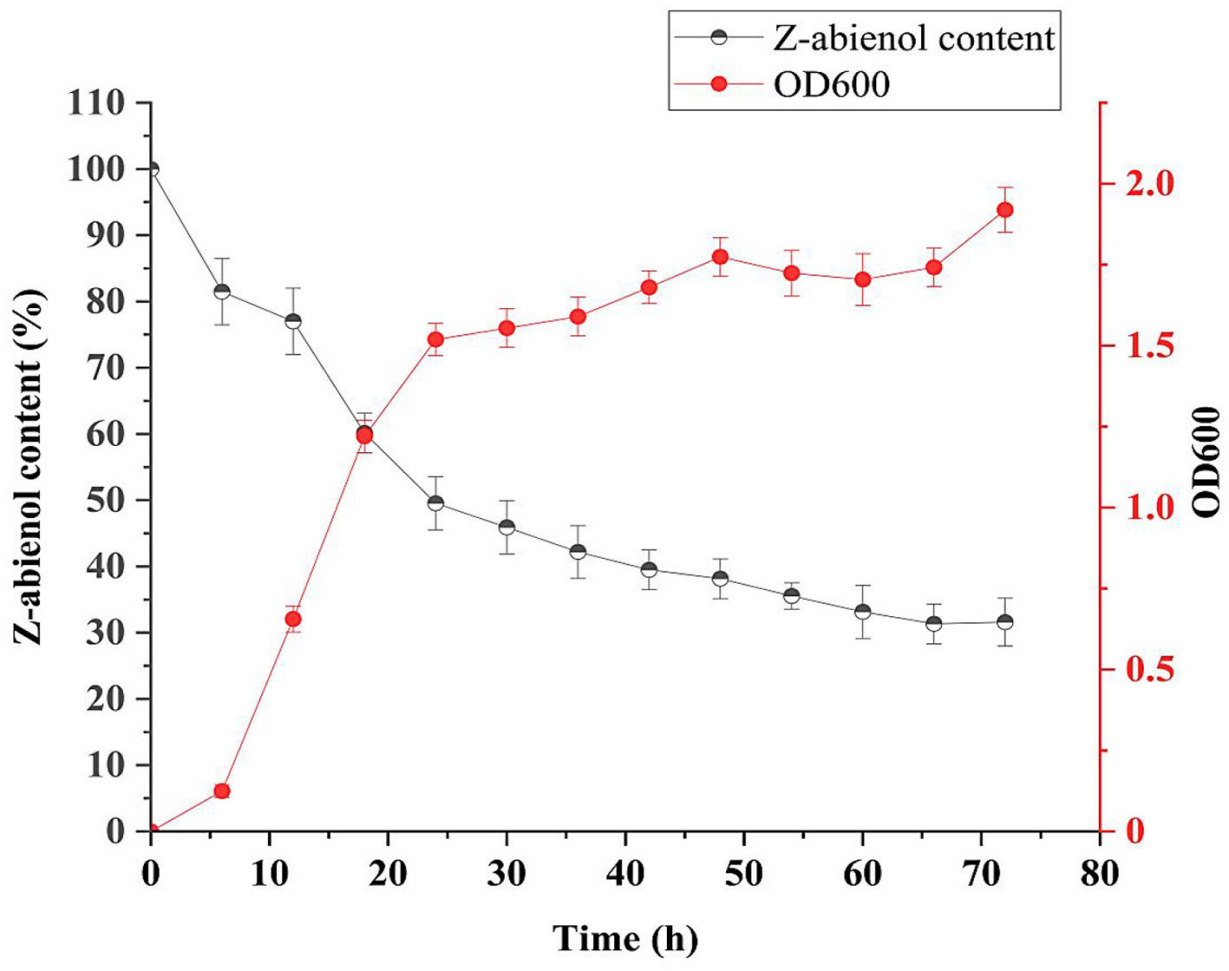
Growth curve and degradation curve of LSC-2. The optical density at 600 nm (OD600) represents the bacterial growth, while the Z-abienol content (%) indicates degradation efficiency over time. The LSC-2 strain shows an initial adaptation phase, followed by active degradation of Z-abienol during the logarithmic and stationary phases. The degradation rate is highest in the first 24 h and gradually decreases, stabilizing after 48 h. Error bars represent standard deviations from triplicate experiments. Values are mean ± standard deviation (SD, *n* = 3).

As represented in Figure 4, growth curve analysis of *A. tjernbergiae* LSC-2 showed a delayed growth phase during the first 0–6 h, which is attributable to the absence of a preferred carbon source, such as glucose. Previous studies have reported similar adaptation periods in microbial strains that metabolize hydrophobic compounds as the bacteria need to activate specific metabolic pathways and transport mechanisms to utilize these substrates efficiently (Ruiz et al., 2010; Sandberg et al., 2017).

Once adaptation occurred, the LSC-2 strain rapidly degraded Z-abienol between 6 and 24 h, which indicates peak metabolic activity during the logarithmic phase. The sharp decline in the Z-abienol content from ~90% to 40% suggests efficient enzymatic breakdown, probably involving oxygenases and dehydrogenases (Sandberg et al., 2017). Furthermore, several studies have reported the biotransformation potential of *Acinetobacter* species in degrading a wide range of compounds (DiMarco et al., 1993; Geissdorfer et al., 1995; Kiran et al., 2013; Lee et al., 1999; Ratajczak et al., 1998).

**Figure 5:**
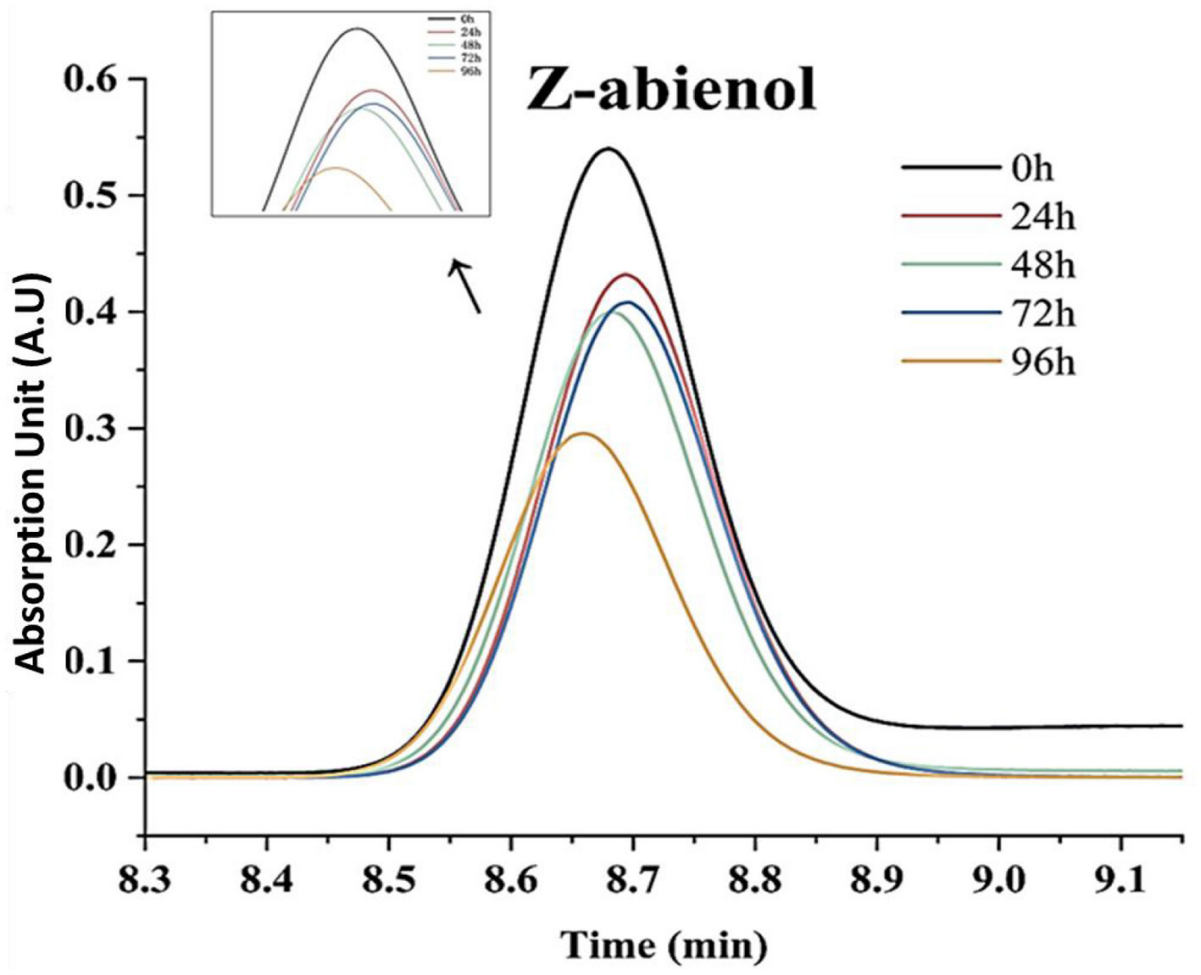
Content variation of Z-abienol at different fermentation times. Chromatographic profiles illustrate the degradation trend of Z-abienol over 96 h, with peak intensity decreasing progressively from 0 h (black) to 96 h (orange). Retention time remains consistent at ~8.7 min, indicating that the compound is structurally stable while undergoing metabolic transformation. The inset highlights the same trend, reinforcing the observed decline in Z-abienol concentration over time. Y-axis represents absorbance (AU), a standard unit in liquid chromatography. AU values are indirectly correlated with component concentrations in the sample.

Z-abienol degradation efficiency of *A. tjernbergiae* LSC-2 was evaluated at 0, 24, 48, 72, and 96 h of fermentation (Figure 5, Table 1). At 24 h, it reached 50.4%, which further increased to 69.3% at 96 h. The progressive decline in peak intensity over time indicates the active degradation of Z-abienol and is consistent with the exponential growth phase of *A. tjernbergiae* LSC-2 (Figures 4, 5).

**Table 1:** Z-abienol degradation efficiency by LSC-2 and metabolite formation.

**Time (h)**	**Z-abienol (μg/mL)**	**Degradation rate (%)**	**Sclareol (μg/mL)**	**Sclaral (sclareolide lactol) (μg/mL)**	**Amberonne (isomer 3) (μg/mL)**
0	1149.7	–	310.6	2.5	6.7
24	561.4	50.4%	168.9	1.6	7.4
48	428.8	61.8%	151.7	4.7	4.8
72	551.2	51.4%	218.7	1.6	5.6
96	341.5	69.3%	211.3	3.2	9.1

“–” indicates no detection.

**Figure 6:**
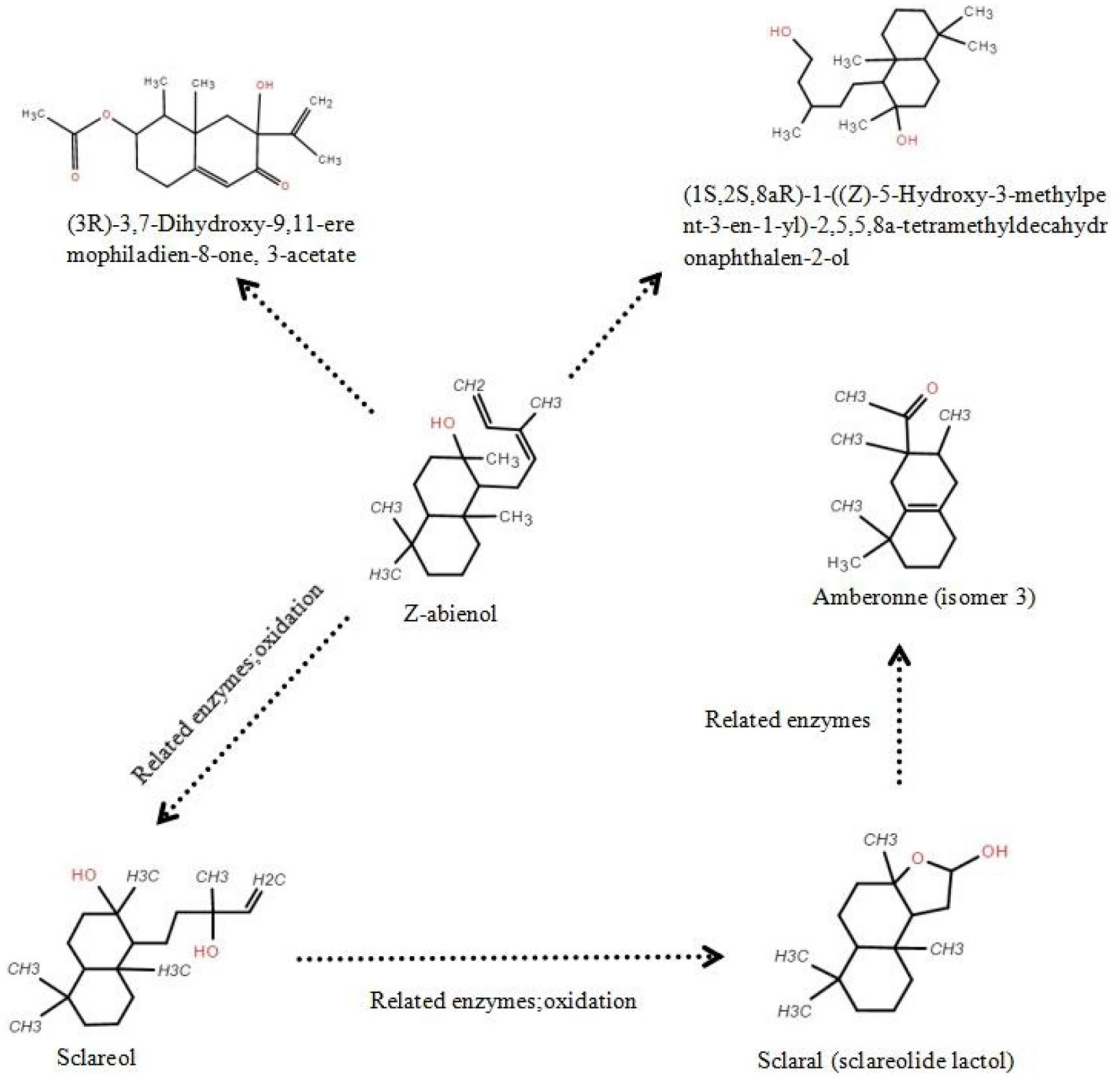
Putative Z-abienol degradation pathway in *A. tjernbergiae* LSC-2 and key enzymes. Hydroxylation of Z-abienol by terpene hydroxylase produces sclareol, which undergoes oxidation via alcohol dehydrogenase to form scalaral (sclareolide lactol). Further oxidation by aldehyde dehydrogenase leads to amberonne, a transient intermediate. In addition, hydroxylation and acetylation steps catalyzed by cytochrome P450 monooxygenase and acetyltransferase result in eremophiladienone derivatives. These transformations suggest a multistep enzymatic pathway enabling diterpene degradation in *A. tjernbergiae* LSC-2.

As shown in Figure 6, GC-MS analysis detected multiple intermediates, including sclareol, scalar (sclareolide lactol), and amberonne, in the proposed degradation pathway of Z-abienol. The enzymatic oxidation of Z-abienol appeared to proceed through hydroxylation and lactonization reactions, forming transient intermediates. Among them, sclareol was detected in the highest concentration, fluctuating over time, with its lowest concentration observed at 48 h (151.7 μg/mL) and partial accumulation at 96 h (211.3 μg/mL). Scalaral and amberonne were detected at lower concentrations, suggesting their transient nature as metabolic intermediates rather than stable end products.

Since no light-proof treatment was employed during fermentation and Z-abienol degradation processes by the *A. tjernbergiae* LSC-2 strain, the degradation of amberonne may be attributable to photolytic cleavage. When exposed to light, particularly ultraviolet radiation, a photolysis reaction can take place, wherein light energy excites chemical bonds within the amberonne molecule, leading to their cleavage. In particular, carbon–carbon or carbon–hydrogen bonds may break under exposure to sufficient energy, generating reactive free radicals. These radicals subsequently interact with the surrounding molecules, such as oxygen and water, and trigger further oxidative and hydrolytic transformations. This suggests that light-induced degradation and enzymatic metabolism may contribute to the observed reduction in amberonne concentration.

These findings indicate that *A. tjernbergiae* LSC-2 follows a multistep enzymatic pathway for diterpene catabolism. The presence of hydroxylated and lactonized derivatives suggests the activity of terpene hydroxylases and lactone-forming enzymes, which have been previously identified in other terpene-degrading bacteria (Martin and Mohn, 2000; Rogowska-van der Molen et al., 2023; Shukla and Beran, 2020). Further transcriptomic and proteomic studies can help elucidate the mechanisms of specific enzymes involved in this pathway (Wang R. et al., 2024) and provide deeper insights into the metabolic capabilities of *A. tjernbergiae* LSC-2.

### 3.3 Effect of growth factors on the degradation of Z-abienol in the growth of *A. tjernbergiae* LSC-2

#### 3.3.1 Effect of Z-abienol concentration

**Figure 7:**
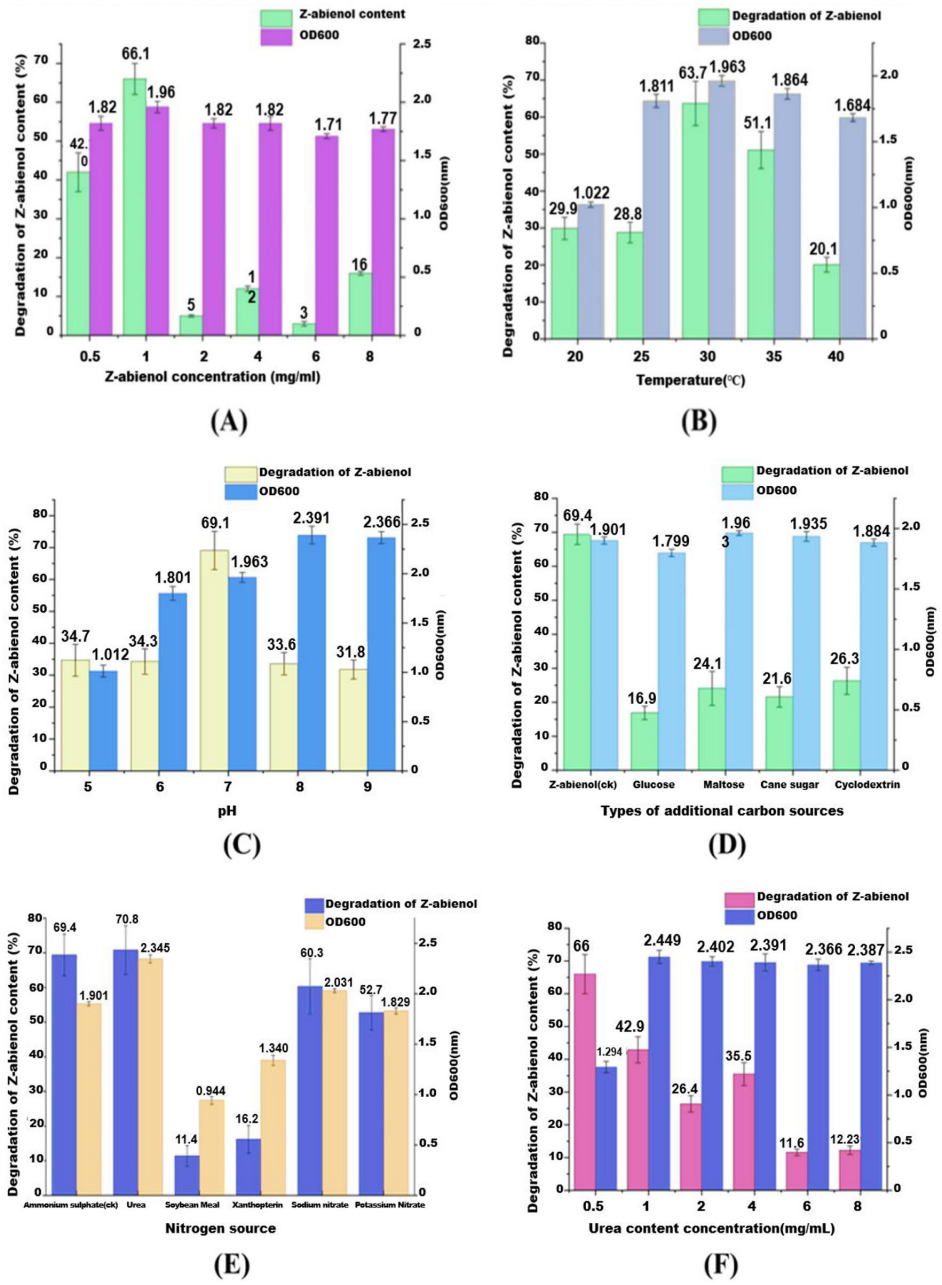
Influence of various environmental factors on the degradation rate of Z-abienol by the LSC-2 strain and microbial growth, as indicated by OD600 values. **(A)** Shows the effect of Z-abienol concentration, where the highest degradation was observed at 1 mg/mL, while a decline was observed at higher concentrations. **(B)** Shows temperature variations, with an optimal degradation of 63.7% at 30°C, suggesting favorable microbial activity at this temperature. **(C)** Shows the effect of pH, where pH 7 resulted in the maximum degradation (69.1%), indicating a preference for neutral conditions. **(D)** Shows the impact of different carbon sources, revealing that maltose significantly enhances degradation (51.1%) compared with glucose, cane sugar, and cyclodextrin. **(E)** Shows nitrogen sources, where urea and ammonium sulfate contributed to the highest degradation (70.8%), while soybean meal had the least effect. **(F)** Shows the influence of urea concentration, showing an optimal degradation at 2 mg/mL, whereas higher concentrations led to reduced efficiency. Collectively, these results highlight the critical role of environmental parameters in optimizing Z-abienol degradation through microbial metabolism. Values are mean ± standard deviation (SD, *n* = 3).

The dose-dependent effects of Z-abienol (0.5–8 mg/mL) on the biodegradation dynamics of *A. tjernbergiae* LSC-2 were systematically investigated. In Figure 7A, green and purple bars depict residual Z-abienol percentage and bacterial growth (OD600), respectively, revealing a non-linear relationship between substrate concentration and metabolic performance. Even though 0.5 mg/mL achieved 55.8% degradation, the optimal concentration was 1 mg/mL with peak degradation efficiency (66.0%) and sustained microbial growth. However, substrate concentrations higher than 1 mg/mL induced significant metabolic suppression, as evidenced by the sharp decline in degradation rates (47.3% at 2 mg/mL) concomitant with growth inhibition patterns.

This biphasic response is consistent with the reported microbial stress responses to lipophilic compounds. Previous studies have documented terpene-induced toxicity thresholds in bacterial systems (Rogowska-van der Molen et al., 2023), and increased substrate concentrations (>1% w/v) are shown to disrupt membrane integrity and enzyme kinetics in γ-proteobacteria (Huang A. et al., 2022; Maduka and Okpokwasili, 2016; Marrot et al., 2006). The observed 35% efficiency drop between 1 and 2 mg/mL concentrations suggests the possible phase partitioning effects or catabolite repression mechanisms specific to *A. tjernbergiae*. Future investigations using proteomic profiling and real-time qPCR need to delineate whether this inhibition stems from (i) the downregulation of diterpenoid catabolism genes, (ii) accumulation of cytotoxic intermediates, or (iii) osmotic stress from hydrophobic compound accumulation.

#### 3.3.2 Effect of temperature

The effect of temperature on Z-abienol degradation by the *A. tjernbergiae* LSC-2 strain was evaluated by cultivating the strain in a fermentation medium supplemented with 1 mg/mL Z-abienol at varying temperatures (20°C−40°C). As shown in Figure 7B, Z-abienol degradation was significantly influenced by temperature. At 20°C and 25°C, it remained relatively low at 29.9% and 28.8%, respectively, with moderate bacterial growth (OD600 = 1.022 and 1.811). The highest degradation rate of Z-abienol (63.7%) was achieved at 30°C, corresponding to the highest bacterial growth (OD600 = 1.963). This suggests that 30°C provides the most favorable conditions for enzymatic activity and degradation of Z-abienol by the *A. tjernbergiae* LSC-2 strain (Ahmad et al., 2011; Luo et al., 2013).

At 35°C, degradation efficiency declined to 51.1% despite a high OD600 value (1.864). This indicates that even though the bacterial strain remained metabolically active, the enzymes involved in Z-abienol degradation might be less efficient at this temperature. A further increase to 40°C resulted in a drastic reduction in degradation efficiency (20.1%) and bacterial growth (OD600 = 1.684), probably due to thermal stress affecting cellular functions and enzyme stability (Ahmad et al., 2011; Luo et al., 2013).

The findings suggest that Z-abienol biotransformation is highly temperature dependent, with excessive heat potentially leading to protein denaturation or metabolic stress. Based on these findings, 30°C was selected as the optimal temperature for all subsequent fermentation experiments to maximize degradation efficiency while maintaining robust bacterial growth.

#### 3.3.3 Effect of pH

Z-abienol degradation efficiency of *A. tjernbergiae* LSC-2 was significantly influenced by pH (Figure 7C). The strain showed moderate Z-abienol degradation efficiency under acidic conditions (pH 5 and 6), with degradation rates of 34.7% and 34.3%, respectively. The bacterial growth, measured as OD600, was also relatively low at these pH levels (1.012 and 1.801, respectively) (Figure 7C). The low degradation efficiency under acidic pH conditions suggests that the enzymes of the metabolic pathway responsible for Z-abienol metabolism are less efficient under these conditions, possibly due to enzyme instability or impaired cellular metabolism (Li et al., 2022).

The highest degradation rate (69.1%) was observed at pH 7, which was correlated with an OD600 value of 1.963, indicating optimal bacterial growth. This suggests that neutral pH conditions provide the most favorable environment for microbial proliferation and enzymatic activity during Z-abienol degradation by *A. tjernbergiae* LSC-2 (Hao et al., 2019).

Beyond pH 7, Z-abienol degradation efficiency declined to 33.6% and 31.8% at pH 8 and 9, respectively, despite higher OD600 values (2.391 and 2.366). This suggests that even though the bacterial strain remained metabolically active, perhaps alkaline conditions altered enzyme conformation or affected membrane transport mechanisms, thus reducing Z-abienol degradation efficiency (Hao et al., 2019; Rogowska-van der Molen et al., 2023). These findings indicate that pH 7 is optimal for Z-abienol degradation by *A. tjernbergiae* LSC-2.

#### 3.3.4 Effect of carbon source

The dependence of the degradation efficiency of *A. tjernbergiae* LSC-2 on the carbon source was quantified through degradation assays with Z-abienol as the target substrate (Figure 7D). As the sole carbon source, Z-abienol supported both peak degradation efficiency (69.4%) and substantial bacterial growth (OD600 = 1.901). In addition, co-supplementation with glucose, maltose, cane sugar, and cyclodextrin significantly suppressed Z-abienol degradation to 16.9%, 24.1%, 21.6%, and 26.3%, respectively, while maintaining high biomass accumulation (OD600 >1.8 across all conditions). This inverse relationship between degradation efficiency and alternative carbon source availability indicates a classic carbon catabolite repression (CCR) response, wherein the preferential utilization of readily metabolizable substrates inhibits secondary catabolic pathways (Galinier, 2018; Görke and Stülke, 2008). The findings confirm glucose as the most potent CCR inducer, which reduces Z-abienol degradation by 75.6% compared with the substrate-only control.

#### 3.3.5 Effect of nitrogen source

The effect of different nitrogen sources on Z-abienol degradation by the *A. tjernbergiae* LSC-2 strain was investigated under the condition of 1 mg/mL Z-abienol concentration at 30°C. The nitrogen sources tested included ammonium sulfate ((NH4)_2_SO4), urea, soybean meal, xanthopterin, sodium nitrate, and potassium nitrate (Figure 7E). Among these, urea showed the highest Z-abienol degradation rate (68.7%) and the highest bacterial growth (OD600 = 2.1), which suggests that urea provides a readily assimilable nitrogen source that enhances the microbial activity and enzymatic processes involved in Z-abienol degradation. This observation is consistent with the findings of previous studies demonstrating that urea addition can stimulate microbial degradation of hydrocarbons. For instance, a previous study has shown that urea application in contaminated soils boosts the biodegradation of hydrocarbons, which indicates the effectiveness of urea in enhancing microbial remediation processes (Keayiabarido and Tanee, 2023). Ammonium sulfate also resulted in significant degradation, but at a lower rate compared with urea. In addition, sodium and potassium nitrate showed moderate degradation rates of 47.8% and 46.9%, respectively, with OD600 values of ~1.85. Soybean meal and xanthopterin showed the lowest degradation efficiencies, 50.2% and 42.5%, respectively, which indicates their relatively lower bioavailability as nitrogen sources for the strain (Figure 7E). These findings suggest that the selection of the nitrogen source is crucial for optimizing the microbial degradation of Z-abienol.

#### 3.3.6 Optimal dose of urea

The effect of varying urea concentrations on Z-abienol degradation by the *A. tjernbergiae* LSC-2 strain was investigated. At a urea concentration of 0.5 mg/mL, the LSC-2 strain showed the highest Z-abienol degradation rate of 66% (Figure 7F). However, as urea concentration increased beyond this point, a decline in degradation efficiency was observed. Notably, at a urea concentration of 1 mg/mL and higher, OD600 measurements showed a significant increase in bacterial growth, approximately twice that of cultures without urea supplementation (Figure 7F). This suggests that the LSC-2 strain may preferentially utilize urea as a carbon and nitrogen source, which reduces its reliance on Z-abienol as the primary carbon source. Thus, higher urea concentrations did not enhance Z-abienol degradation. These findings are in agreement with those of previous research, indicating that some microorganisms can utilize urea as both a carbon and nitrogen source.

For instance, studies have shown that microbial ureases hydrolyze urea to ammonia and carbon dioxide, which can then be assimilated via cellular metabolism (Krausfeldt et al., 2019; Mobley and Hausinger, 1989).

### 3.4 Whole-genome sequence analysis of *A. tjernbergiae* LSC-2

The sequencing of *A. tjernbergiae* LSC-2, generated using Illumina technology, yielded a total of 7,502,484 reads. The percentage of ambiguous bases was minimal at 0.0422%, and the GC content was 39.07%. Notably, 99.11% of the bases had a base-calling accuracy exceeding 99% (Q20), and 97.48% of the bases surpassed 99.9% accuracy (Q30) (Table 2).

**Table 2:** Summary of sequencing data for strain LSC-2.

**Sample name**	**Total reads**	***N* (%)**	**GC content (%)**	**Q20 (%)**	**Q30 (%)**
LSC-2	7,502,484	0.0422	39.07	99.11	07.48

These data indicate high-quality sequencing data. In Illumina sequencing, quality scores (Q scores) are logarithmically related to the probability of incorrect base calls. A Q20 score corresponds to a 1% error rate, whereas a Q30 score corresponds to a 0.1% error rate. High Q scores are essential for reliable downstream analyses as lower scores can lead to high false-positive variant calls and inaccurate conclusions (Bejaoui et al., 2025; Kastanis et al., 2019).

### 3.5 Analysis of the carbohydrate-active enzyme lines (CAZymes)

The molecular structure of Z-abienol comprises multiple functional groups, including hydroxyl (–OH) groups and carbon–carbon double bonds, which are highly susceptible to oxidation. Oxidases can introduce structural and functional modifications in Z-abienol in biological systems. For instance, hydroxyl groups can undergo oxidation to form carbonyl (–C=O) groups, whereas carbon–carbon double bonds may either cleave or participate in addition reactions. This progressive transformation facilitates the conversion of Z-abienol into sclaral through a cascade of oxidation–reduction (redox) reactions, a process predominantly driven by oxidoreductase enzymes [auxiliary activity (AA) enzymes] (Bicas et al., 2009; Sultana and Saify, 2013; Zhou et al., 2024).

**Figure 8:**
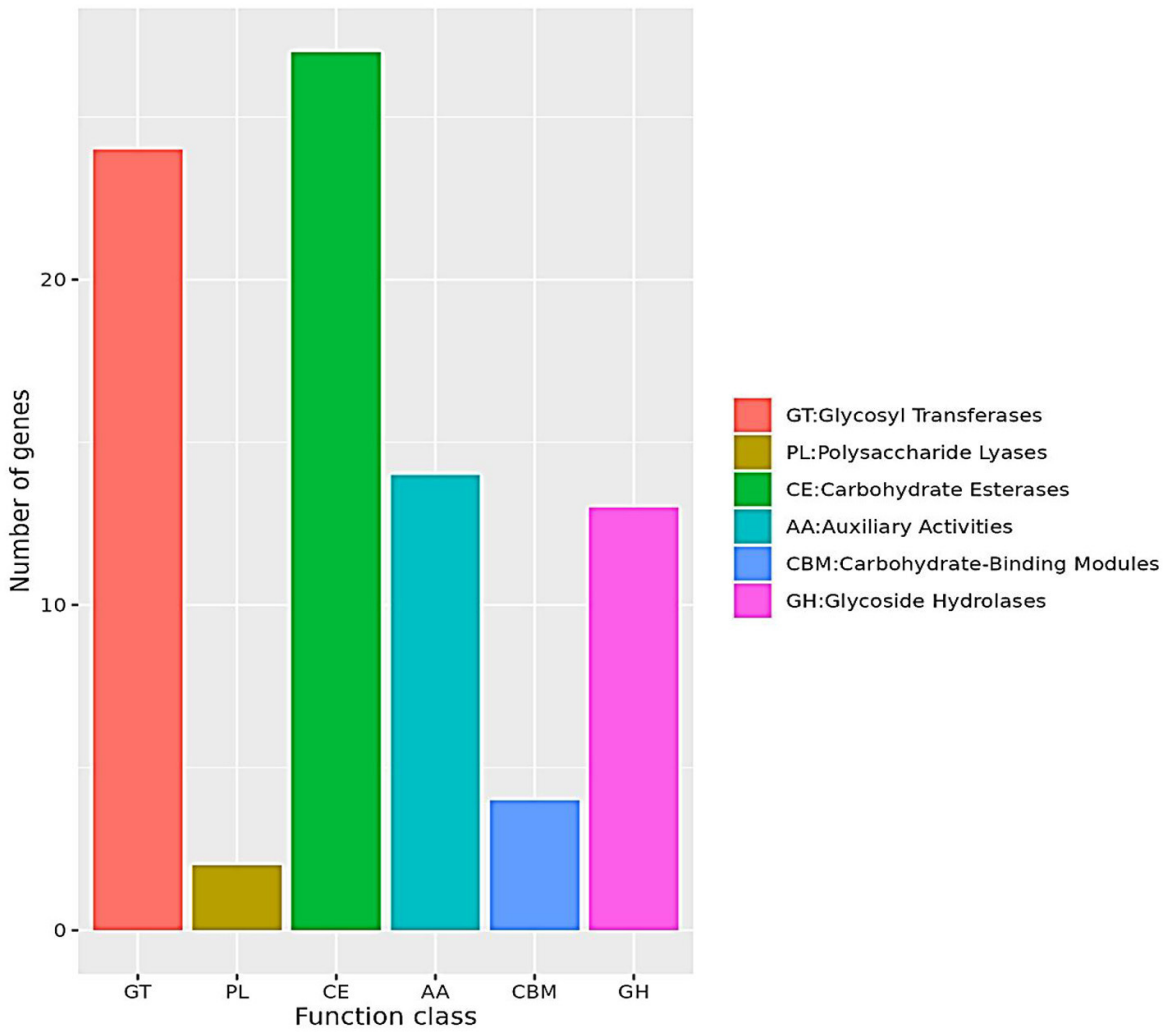
CAZy functional classification. The bar chart represents the classification of Carbohydrate-Active enZymes (CAZymes) based on their functional categories. The x-axis denotes different CAZyme classes, including glycosyl transferases (GT), polysaccharide lyases (PL), carbohydrate esterases (CE), auxiliary activities (AA), carbohydrate-binding modules (CBM), and glycoside hydrolases (GH). The y-axis indicates the number of genes associated with each function class. The chart shows that CE and GT have the highest gene counts, whereas PL has the lowest. The color-coded legend provides a clear distinction between the CAZyme categories.

Comparative gene distribution in the genome of the LSC-2 strain is presented in Figure 8. Specifically, the functional classification of CAZymes in LSC-2 includes 27 glycosyltransferase (GT) genes, 24 glycosyl hydrolase (GH) genes, 14 AA oxidoreductases, 2 polysaccharide lyase genes, and 4 carbohydrate-binding module genes. The dominance of AA oxidoreductases among these CAZymes suggests their crucial role in the oxidative degradation of Z-abienol. The carbohydrate-degrading enzymes in LSC-2 are categorized into six major classes, among which AA oxidoreductases are the most likely candidates involved in Z-abienol degradation. The presence of 14 AA oxidoreductases implies a potential enzymatic pathway for Z-abienol breakdown, possibly involving laccases, peroxidases, or monooxygenases, which have been documented to catalyze similar oxidation–reduction reactions in plant-derived terpenoids (Bicas et al., 2009; Dixit et al., 2021; Kuriata-Adamusiak et al., 2012; Malhotra and Suman, 2021; Martínez et al., 2017; Nowrouzi and Rios-Solis, 2022; Singh et al., 2023; Spyrou et al., 2023).

Furthermore, GHs and GTs may modify the glycosidic linkages of intermediate metabolites during Z-abienol degradation, thus facilitating their subsequent oxidation. Studies have shown that CAZyme families, especially AA3 (glucose–methanol–choline oxidoreductases) and AA7 (glucoside oxidases), play a vital role in terpene oxidation and degradation (Roy et al., 2020; Zhou et al., 2024). These findings support the hypothesis that LSC-2 can metabolize Z-abienol through a coordinated enzymatic mechanism involving multiple CAZymes, particularly oxidoreductases.

The functional classification of CAZymes in LSC-2 reveals the metabolic potential for the enzymatic degradation of Z-abienol. The significant presence of AA oxidoreductases, alongside GTs and GHs, suggests a complex oxidation–reduction pathway that facilitates Z-abienol conversion. Further biochemical and genetic analyses (Wen et al., 2024) are needed to elucidate the precise molecular mechanisms of Z-abienol degradation in LSC-2.

## 4 Conclusion

This study identified and characterized *A. tjernbergiae* LSC-2 as a bacterial strain capable of efficiently degrading Z-abienol, a diterpenoid found in tobacco leaves. Phylogenetic analysis based on 16S rDNA sequencing confirmed its taxonomic placement within the *Acinetobacter* genus. Using Z-abienol as the sole carbon source, LSC-2 showed an optimal degradation efficiency of 69.3% under optimized fermentation conditions (30°C, pH 7, 150 rpm, 4 days). The enzymatic breakdown of Z-abienol was facilitated by oxidoreductases, primarily from the AA family, as suggested by whole-genome sequencing and functional gene annotation. Furthermore, the findings of this study showed that external factors, such as temperature, pH, and nitrogen source, significantly influence degradation efficiency, with urea (0.5 mg/mL) being the most suitable nitrogen source. The metabolic pathway is shown to be involved in hydroxylation and oxidation reactions, leading to the formation of degradation intermediates such as sclareol, sclareolide lactol, and amberonne.

These findings highlight the potential of *A. tjernbergiae* LSC-2 in biotransformation applications, particularly in the tobacco industry, where microbial degradation can enhance the production of aroma compounds. Future studies should focus on elucidating enzymatic pathways and metabolic engineering approaches to improve the efficiency and scalability of this bioconversion process.

## Data Availability

16S rDNA sequence of A. tjernbergiae LSC-2 strain was submitted to the NCBI Sequence Read Archive (https://www.ncbi.nlm.nih.gov) with the accession number PV065465.
