# Supplementary figures and images for: Biotransformation of tobacco-derived Z-abienol into precursors of ambrox by newly identified Acinetobacter tjernbergiae LSC-2
Source: Front Microbiol. 2025 May 14;16:1581788. doi: 10.3389/fmicb.2025.1581788 (PMC12117261; doi:10.3389/fmicb.2025.1581788)

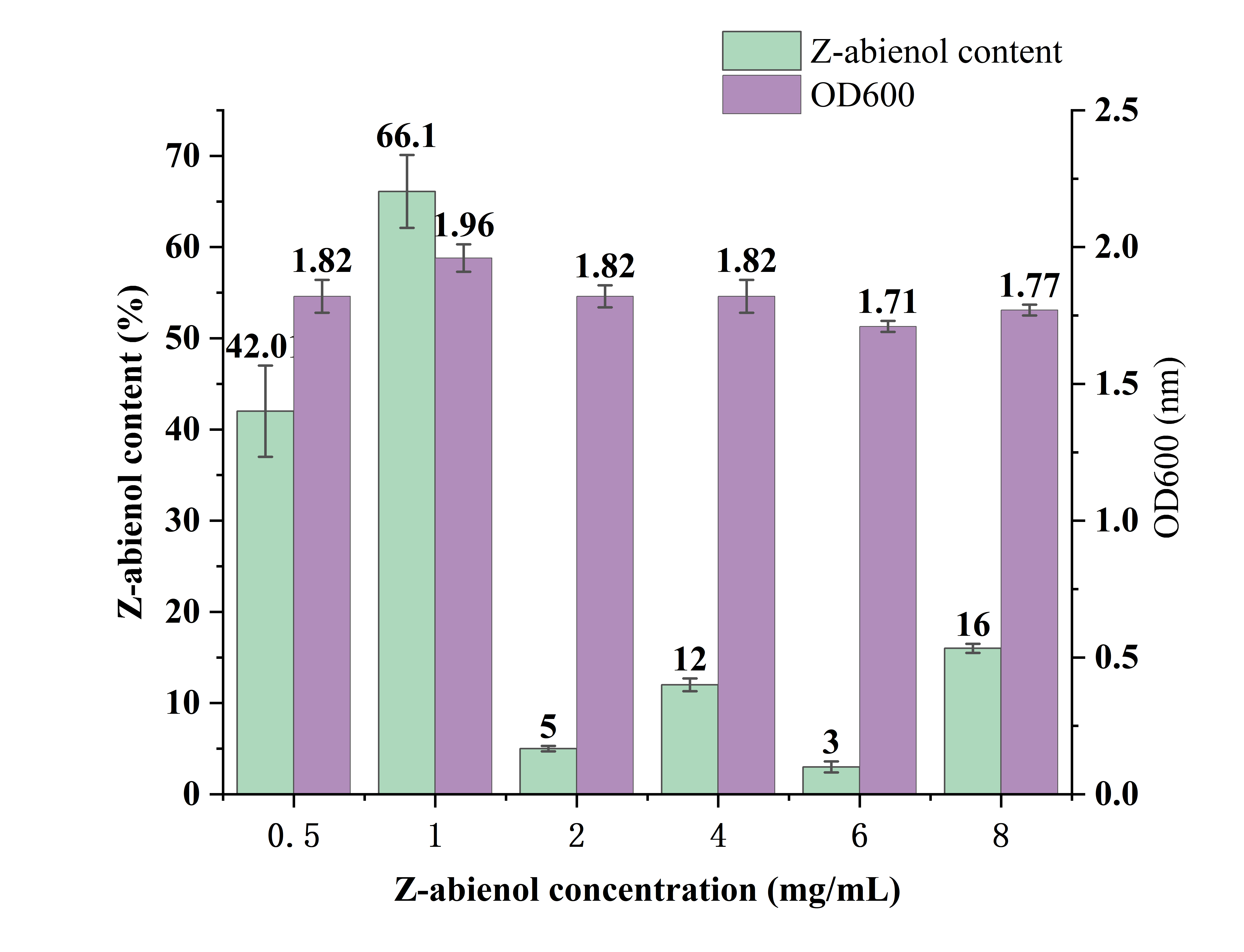

Supplement: Supplementary file 1 [file Image_1.jpg]

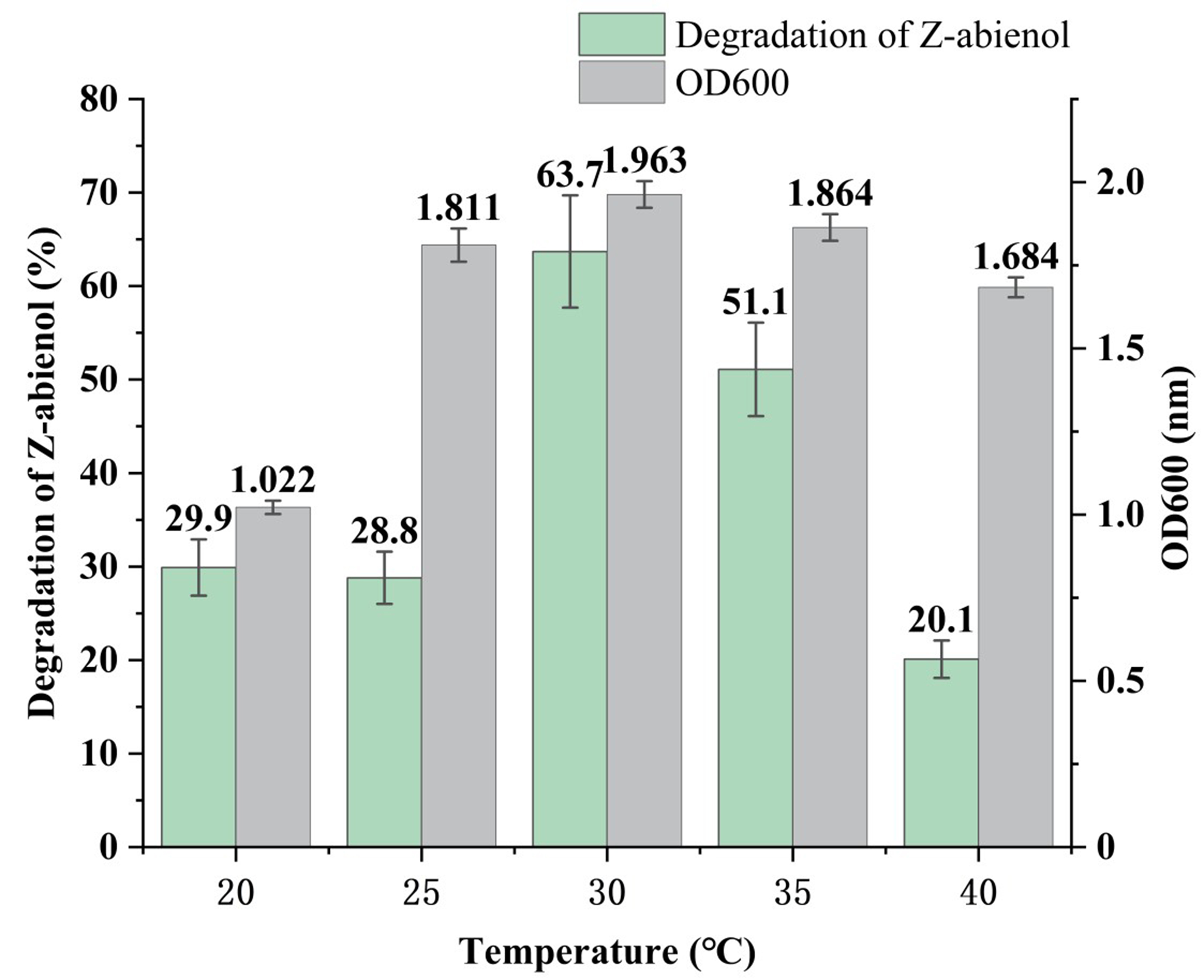

Supplement: Supplementary file 2 [file Image_2.jpeg]

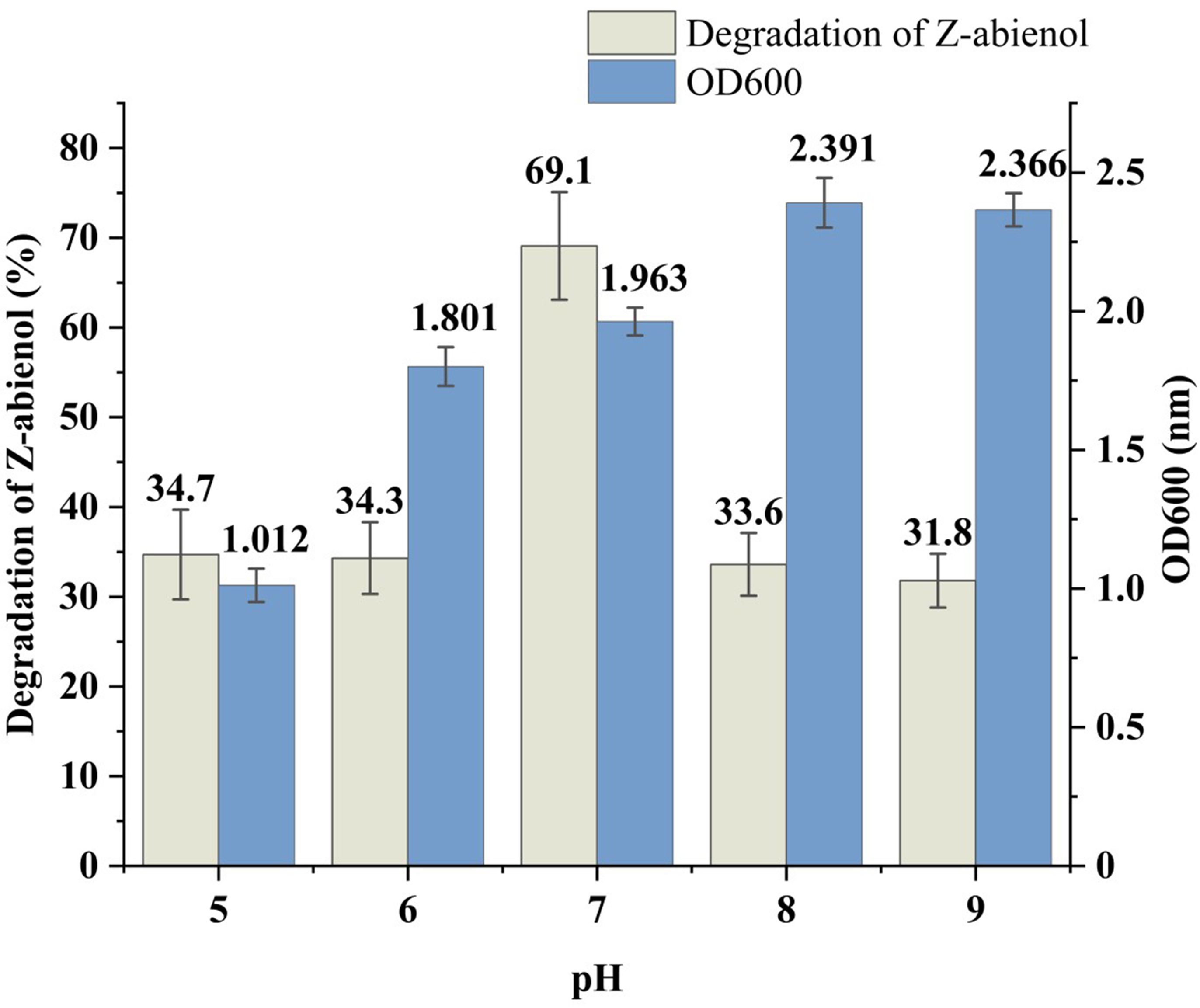

Supplement: Supplementary file 3 [file Image_3.jpeg]

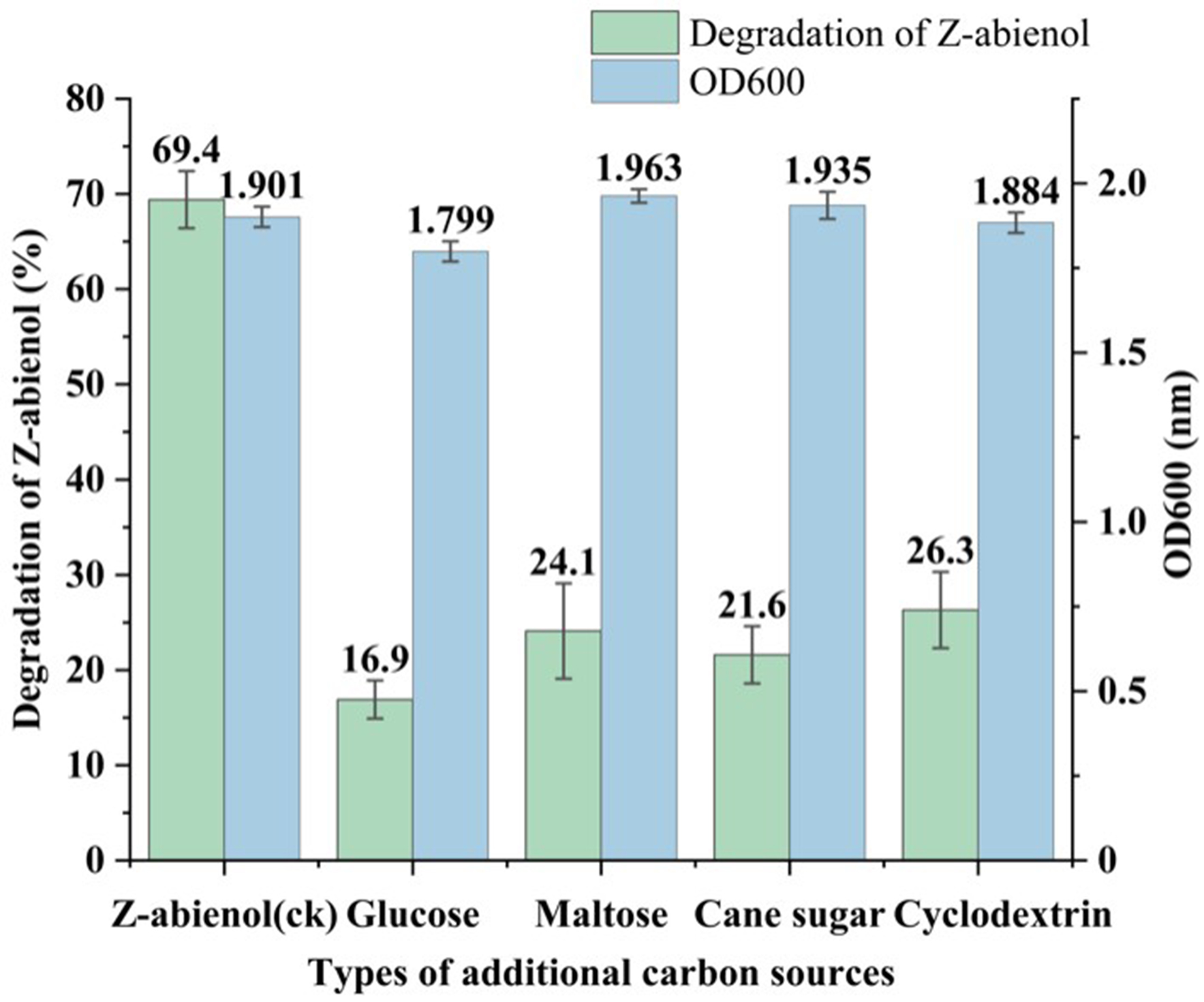

Supplement: Supplementary file 4 [file Image_4.jpeg]

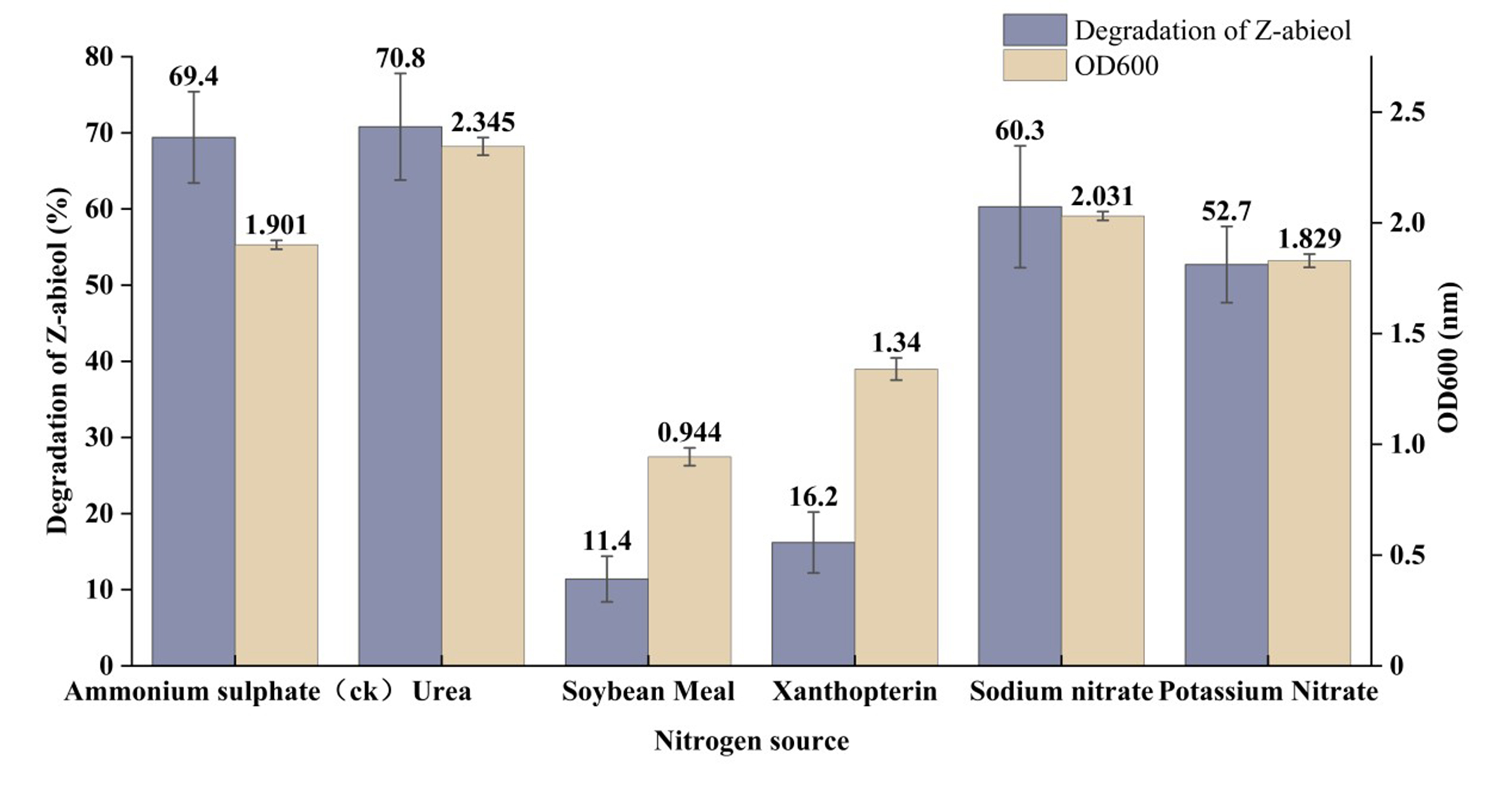

Supplement: Supplementary file 5 [file Image_5.jpeg]

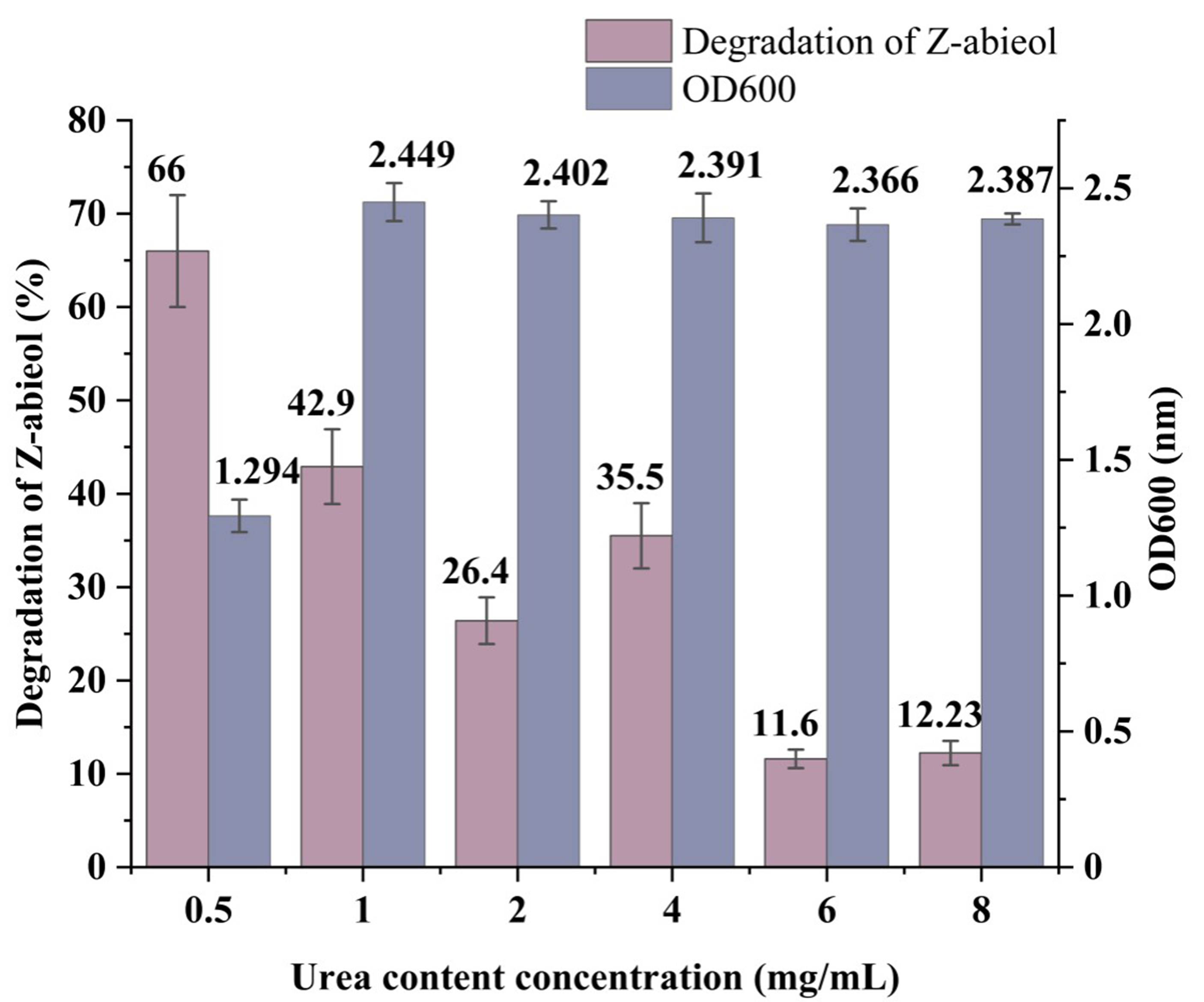

Supplement: Supplementary file 6 [file Image_6.jpeg]
